# Mitochondria affect antibacterial immunity in cross talk with innate immune response molecules coded by genes of nuclear origin

**DOI:** 10.3389/fimmu.2026.1918926

**Published:** 2026-09-21

**Authors:** Argha Chakraborty, Aruna Pal, Manti Debnath, Jyoti Sahu, Abantika Pal, Joydeep Mukherjee, Rajarshi Samanta, Paresh Nath Chatterjee

**Affiliations:** 1 Department of Animal Genetics and Breeding, Kolkata, West Bengal, India; 2 West Bengal University of Animal and Fishery Sciences, Kolkata, India; 3 Department of Livestock Farm Complex, Kolkata, West Bengal, India; 4 Department of Livestock Production and Management, Kolkata, West Bengal, India; 5 University of California, San Francisco, San Francisco, CA, United States; 6 Department of Veterinary Physiology, Kolkata, West Bengal, India; 7 Department of Fish Nutritionl, Kolkata, West Bengal, India

**Keywords:** *Anas platyrhnchos*, antibacterial immunity, duck pasteurellosis, mitochondrial genome, nuclear gene

## Abstract

**Introduction:**

Mitochondria are dynamic, semi-autonomous organelles that, in addition to their central role in ATP production, regulate calcium homeostasis, iron metabolism, apoptosis, and innate immune signalling. Although the mitochondrial genome encodes only 37 genes, mitochondrial architecture, bioenergetic function, and dynamics are governed predominantly by nuclear-encoded proteins, necessitating highly coordinated communication between mitochondrial DNA (mtDNA) and the nuclear genome. Disruption of this bidirectional mitochondrial–nuclear crosstalk has been increasingly implicated in the pathogenesis of numerous diseases across species; however, the molecular mechanisms underlying this communication remain incompletely understood.

**Materials and method:**

In the present study, we used *Anas platyrhynchos* as a model of duck pasteurellosis to investigate mitochondrial–nuclear interactions in the regulation of innate immune responses during *Pasteurella multocida* infection. We characterized the mitochondrial (cytochrome *b* and cytochrome *c*) and nuclear genes (NLRP3, IL-18, and STING) of duck, in silico molecular docking, PHYRE 2 for PDB structure prediction, pymol visualization, and experimental validation with differential mRNA expression profiling.

**Result and discussion:**

Our findings demonstrate that the mitochondrially encoded proteins cytochrome b and cytochrome c functionally interact with the nuclear-encoded immune regulators NLRP3, IL-18, and STING through the NOD-like receptor signalling pathway, phagocytic pathways, and the cytosolic DNA-sensing pathway. Furthermore, molecular docking analyses identified putative interaction sites between IL-18 and *P. multocida*, providing additional support for the functional relevance of these immune pathways during bacterial infection. To our knowledge, this study provides the first evidence in an animal model that mitochondrial retrograde signalling influences the regulation of nuclear immune genes during bacterial infection. These findings reveal a previously unrecognized mechanism through which mitochondrial gene products modulate host innate immune responses, thereby advancing our understanding of mitochondrial–nuclear communication in host–pathogen interactions.

## 1 Introduction

Host immunity is a fundamental determinant of host–pathogen interactions, encompassing a complex and dynamic network of biological processes that govern the interplay between invading pathogens and the host throughout the course of infection. The innate immune system serves as the first line of defense and is orchestrated through a highly coordinated cascade of signaling molecules, immune mediators, and effector proteins, the expression and activity of which are tightly regulated by immune-associated genes. These genes play critical roles in host defense by mediating pathogen recognition through pattern recognition receptors (PRRs) and regulating the production of pro-inflammatory and anti-inflammatory cytokines, thereby shaping the magnitude, duration, and outcome of the immune response.

Our laboratory has previously demonstrated the pivotal role of specific nuclear genes in regulating disease resistance and overall health across multiple livestock and poultry species, including sheep (1, 2), cattle (3), buffalo (4), goats (5, 6), chickens (7), and ducks (8, 9), using established animal models (10). In parallel, we have elucidated the contribution of mitochondrial genes to host health, disease resistance (11), and reproductive performance (12). Although mitochondria are traditionally recognized for their central role in oxidative phosphorylation and cellular energy production, accumulating evidence indicates that mitochondrial genes also function as key regulators of a wide range of cellular processes, including calcium homeostasis (13), iron metabolism (14), redox signaling, apoptosis, and innate immune responses. These findings underscore the multifaceted role of mitochondrial function in maintaining cellular homeostasis and orchestrating host defense mechanisms.

Furthermore, our preliminary investigations encompassing all 37 mitochondrial genes in duck, conducted through next-generation sequencing-based complete mitochondrial genome analysis, have provided important insights into mitochondrial genomic organization and functional architecture (15–17). In addition, comprehensive genomic studies in ducks have further advanced our understanding of the genetic determinants underlying key biological traits (18–20). Recent evidence suggests that mitochondrial DNA (mtDNA) functions not only as a genetic component of energy metabolism but also as a critical signaling molecule capable of modulating nuclear-encoded immune response pathways, thereby influencing host defense mechanisms and pathogen clearance (21).

Pasteurellosis is a zoonotic disease of considerable public health importance, affecting humans as well as a broad range of domestic animal species. In poultry, duck cholera caused by *Pasteurella multocida* represents a major bacterial disease associated with high morbidity, mortality, and substantial economic losses to the poultry industry. The chronic form of duck cholera is characterized by persistent colonization of the pathogen within specific tissues and organs, whereas the acute form is associated with severe clinical manifestations, including watery diarrhea, anorexia, respiratory distress, septicemia, and rapid mortality. Fowl cholera continues to impose significant economic constraints on poultry production due to production losses, increased mortality, reduced productivity, and elevated expenditure on therapeutic and preventive interventions. Economic analyses have highlighted the substantial financial burden associated with disease outbreaks. For example, during a fowl cholera outbreak in a flock of 50,000 birds, the estimated vaccination cost was approximately USD 0.40 (EUR 0.33) per bird, whereas routine vaccination in non-outbreak flocks was estimated at USD 0.12 (EUR 0.10) per bird. Furthermore, treatment-related costs during outbreaks between 1985 and 1986 were estimated at USD 0.51 (EUR 0.45) per bird, including productivity losses, with nearly 50% of total expenditure attributed to post-outbreak immunization strategies (22). Overall, fowl cholera accounted for an estimated 0.5% reduction in total turkey meat production in California during the 1985–1986 period, underscoring the substantial economic and production-level impact of *P. multocida* infections. In ducks, *P. multocida*-induced cholera remains a major constraint to production systems, resulting in significant morbidity, mortality, and economic losses. Accordingly, elucidating the genetic and molecular mechanisms underlying host resistance to this bacterial pathogen is essential for the development of improved disease control strategies and enhanced disease resilience in poultry populations.

Ducks are the second most important poultry species after chickens and exhibit several distinctive biological traits, including relatively higher resistance to a range of economically important avian diseases (8). Indigenous duck populations, in particular, display notable disease resilience and environmental adaptability, enabling their sustainable rearing under organized farming systems with minimal water requirements, provided that basic access to water is available for essential behaviors such as head dipping and ocular hygiene. Despite these advantageous traits, ducks remain comparatively underexplored as a poultry model, particularly with respect to the molecular mechanisms governing innate immune regulation and host defense responses.

Certain preliminary studies have been undertaken depicting the role of immune response genes controlling the resistance against pathogens. Similarly, certain reports on the role of mitochondrial gene against pathogenic infection have been studied (21, 23, 24). This is the first report where we are studying the complex interaction or cross talk of mitochondria with nuclear encoded immune response molecule against bacterial infection.

In the present study, we aimed to characterize selected nuclear-encoded innate immune response genes, together with mitochondrial protein-coding genes, and to elucidate their potential functional interactions at the protein level in regulating host immune responses against duck pasteurellosis. The study was conducted using *Anas platyrhynchos* as an experimental model to investigate the molecular basis of host–pathogen interactions and to assess the contribution of nuclear–mitochondrial genomic crosstalk to bacterial disease resistance.

## 2 Materials and methods

### 2.1 Characterization of mitochondrial gene (cytochrome B) & nuclear gene coding for mitochondrial protein-(cytochrome C) and nuclear gene (NLRP3, IL18 and sting) in liver tissue of *Anas platyrynchos*

#### 2.1.1 Birds and sample collection

An outbreak of pasteurellosis occurred naturally in indigenous Bengal ducks (n=60) maintained at the Experimental Duck Farm, Livestock Farm Complex, West Bengal University of Animal and Fishery Sciences, following a natural disaster. Based on the disease outcome, the birds were classified into two groups: (i) an infected group comprising ducks that exhibited characteristic clinical signs of pasteurellosis and subsequently succumbed to the infection, and (ii) a healthy group consisting of asymptomatic survivor ducks that remained clinically normal despite natural exposure during the outbreak. Further step involves molecular confirmation of the infected birds through PCR based detection with Pasteurella specific gene KMT1, while in healthy birds, KMT1 gene could not be detected.

Tissue samples from infected ducks were collected as part of the routine disease diagnostic investigations conducted following the natural outbreak. A presumptive diagnosis of pasteurellosis was established based on clinical manifestations and characteristic post-mortem lesions and was subsequently confirmed by molecular detection of *Pasteurella multocida*. Liver samples from healthy ducks were obtained from a local slaughter facility, where surplus clinically healthy birds originating from the same flock and naturally exposed to the outbreak were processed as part of routine farm management practices. These birds exhibited no clinical signs of pasteurellosis throughout the outbreak period and were therefore considered the healthy control group.

#### 2.1.2 Sample collection and RNA isolation

Our experimental farm consisted of sixty number of ducks, which were naturally exposed to *Pasteurella multocida* infection. Around fifty percent of the ducks were symptomatically infected. We have randomly sampled six ducks from each group, healthy as well as infected. We confirmed the presence of Pasteurella multocida through molecular detection. Ducks detected with *Pasteurella multocida* were considered as infected, while those detected negative with Pasteurella were considered as healthy. Adult male ducks, aged 6 months were considered under present study. All the ducks under study were hatched on same date and were of same age. Bengal ducks, indigenous documented ducks of West Bengal were considered for the present study.

Samples were collected after 3-4 days after the onset of outbreak in the farm. We assume that the susceptible birds with less innate immunity become infected, while the resistant birds with better innate immunity remain healthy. Liver tissue samples (approximately 1 g) were collected from healthy and naturally infected adult male Bengal ducks (n = 6 per group) following a natural outbreak of pasteurellosis. Immediately after collection, the tissues were immersed in TRIzol reagent and transported to the laboratory under cold-chain conditions for subsequent RNA extraction. Total RNA was isolated using the TRIzol-based extraction method (Life Technologies, USA) according to the manufacturer’s instructions and was subsequently used for complementary DNA (cDNA) synthesis as described previously (25–28). The concentration and quality of the synthesized cDNA were assessed, and samples with cDNA concentrations exceeding 1200 ng/µL were selected for subsequent molecular analyses.

In addition to liver, tissue samples from immune-associated and target organs, including gut-associated lymphoid tissue (GALT) at the ileocecal junction, gizzard, caecum, jejunum, spleen, and pancreas, were collected from both healthy and *Pasteurella multocida*-infected ducks (n = 6 per group). Infection with *P. multocida* was confirmed by molecular detection prior to downstream analyses. These tissue samples were subsequently used to investigate tissue-specific expression profiles of immune- and mitochondria-associated genes and to evaluate molecular responses associated with *P. multocida* infection.

As already explained, the asymptomatic birds following the natural exposure or outbreak, further confirmed negative for *Pasteurella multocida* through molecular (PCR based) technique, were considered healthy under current study. As routine farm management procedure, the surplus healthy birds were sold to local slaughter house. For the purpose of our experiment, we have collected such designated healthy ducks from slaughter house.

#### 2.1.3 cDNA synthesis and PCR amplification of the mitochondrial genes and nuclear genes of Bengal duck affecting antibacterial resistance

First-strand cDNA was synthesized in a final reaction volume of 20 μL containing 5 μg of total RNA, 0.5 μg of oligo(dT) primer (16–18 mer), 40 U of RNase inhibitor, 1000 μM dNTP mixture, 10 mM dithiothreitol (DTT), and 5 U of Moloney Murine Leukemia Virus (M-MLV) reverse transcriptase in the appropriate reaction buffer. The reaction mixture was gently mixed and incubated at 37°C for 1 h, followed by enzyme inactivation at 70°C for 10 min. The synthesized cDNA was immediately chilled on ice, and its integrity was verified by polymerase chain reaction (PCR) as described previously (25–28). The concentration and purity of the synthesized cDNA were subsequently determined using a NanoDrop spectrophotometer.

Gene-specific primer pairs for *NLRP3*, *IL18*, *STING*, *Cytochrome b* (*CytB*), and *Cytochrome c* (*CytC*) were designed using DNASTAR software (Hitachi Miraibio Inc., USA) based on the published chicken and duck nucleotide sequences to amplify the full-length open reading frames (ORFs) of the respective genes (**Table 1**).

**Table 1A:** Primer for mitochondrial genes (cytochrome B) of Bengal duck.

Sl no.	Primer	Tm
1.	5′ GCCATTCACTTCCTACTACCCTTTT 3′	60.8
	5′ TGAATGGGTGTTCGACAGGT 3′	59.2
2.	5′ CACTTCCTACTACCCTTTTTAATCGC 3′	60.0
	5′ TGATGAATGGGTGTTCGACA 3′	57.2

PCR amplification was performed in a final reaction volume of 25 μL containing 80–100 ng of cDNA template, 1× PCR assay buffer, 0.5 μL of 10 mM dNTP mixture, 1 U of Taq DNA polymerase, 60 ng of each forward and reverse primer, and 2 mM MgCl_2_. Amplification was carried out in a PTC-200 thermal cycler (MJ Research, USA) under the following cycling conditions: an initial denaturation at 94°C for 3 min; 35 cycles of denaturation at 94°C for 30s, primer annealing at ranging from 54°C -61°C for 35 s, and extension at 72°C for 3 min; followed by a final extension at 72°C for 10 min. The primer sequences and amplification details for each target gene are provided in **Tables 1a**, **b**.

**Table 1B:** Primers for nuclear gene encodes mitochondrial protein cytochrome C, nuclear immune gene encodes NLRP3, IL18, STING in liver tissue of Bengal duck.

Sl no.	Primer for cytochrome C	Tm
1.	5′ GGAGGTCTGATTGGCTAGCG3′	60.3
	5′ TGCATTGCCATCTCCTTTGTG3′	59.5
2.	5′ GACGTGGAGAAGGGCAAGAA3′	60.0
	5′ TCCATAAGTTAGTGGCTGCCA3′	59.1
	Primer for IL18 gene in Duck	
**1.**	F- 5’TGTGCAGAGAGGGAAGGAAG3’	59.02
	R-5’TCTTCAAAAGCTGCCATGTTCA3’	59.04
2.	F-5’ATCTGGCAGCGGAATGAACT3′	59.7
	R-5’ACTGTTTAGTAATCACAGGTTGTACC3′	59.1
	Primer for amplification of NLRP3 gene in duck	
1.	5′ CTGTGATGGCGGGGGAAG3′	60.1
	5′ AGTGTCTGTGCAGTGACAGC3′	60.5
2.	5′ GGCATGGGGAAGACGATGAT3′	59.9
	5′ TCTCATTTCCCCTGGCGAAC3′	60.0
3	5′ CCAGGGGAAATGAGAGCCTC3′	59.8
	5′ TGCACCCGATTCTTTCACCT3′	59.6
4	5′ TGCAGAATTTCACCACGAGC3′	59.1
	5′ CTCCATCGCCCAGCTCCAG3′	62.5
5	5′ TGCAGTGGTGTGGATTGACA3′	59.8
	5′ GATCTTCAGGCTGGGCTTCA3′	59.7
6	5′ CACACCTGGAGGAGCTGGA3′	60.9
	5′ GGTTTCTGTTGCTGGGAGGA3′	59.9
Sl no.	Primer for amplification of Sting gene	Tm
1.	5′ CCAGGACCCGTCAACCAG 3′	59.7
	5′ GTAGTAGGACCAGGCAAGGC 3′	59.8
2.	5′ AAGTAGTTCTGCCACGCCTG 3′	60.3
	5′ TGCACCATATACTCCTCGCG 3′	59.7
3	5′ CCCGGGCAGGCATCAAAA 3′	60.7
	5′ GTCGGAGGAGCTGACCTG 3′	59.1

#### 2.1.4 cDNA cloning and sequencing

PCR-amplified products of the target genes were verified by electrophoresis on a 1% agarose gel. The desired amplicons were excised from the gel and purified using a Gel Extraction Kit (Qiagen GmbH, Hilden, Germany) according to the manufacturer’s instructions. Purified PCR products were subsequently ligated into the pGEM^®^-T Easy cloning vector (Promega, Madison, WI, USA).

For bacterial transformation, 10 μL of the ligation mixture was gently mixed with 200 μL of competent *Escherichia coli* cells and subjected to heat-shock transformation at 42°C for 45 s in a water bath. Immediately after heat shock, the cells were transferred to ice for 5 min, followed by the addition of SOC medium for recovery.

Following recovery, the transformed bacterial culture was centrifuged, and the cell pellet was plated onto Luria–Bertani (LB) agar plates supplemented with ampicillin (100 μg/mL), isopropyl β-D-1-thiogalactopyranoside (IPTG), and 5-bromo-4-chloro-3-indolyl β-D-galactopyranoside (X-Gal) for blue–white colony screening. White recombinant colonies were selected and cultured overnight at 37C, and plasmid DNA was isolated using the small-scale alkaline lysis method as described previously (21).

The presence of recombinant plasmids was confirmed by PCR using gene-specific primers and by restriction enzyme digestion. Recombinant plasmids were digested with EcoRI (MBI Fermentas, USA) to release the inserted gene fragments, which were subsequently sequenced using the dideoxy chain-termination method with T7 and SP6 universal primers on an automated DNA sequencer (ABI Prism; Chromous Biotech, Bangalore, India).

Sequence analysis.

Nucleotide sequence analyses, including protein translation, multiple sequence alignment, contig assembly, and comparative sequence analysis, were performed using DNASTAR software (Version 4.0; DNASTAR Inc., Madison, WI, USA). The resulting nucleotide sequences were submitted to the National Center for Biotechnology Information (NCBI) GenBank database, and the corresponding accession numbers were obtained and made publicly available.

#### 2.1.5 Studies for predicted mitochondrial and nuclear protein *in silico* of *Anas platyrynchos* in relevance to resistance against *Pasteurella multocida*

##### 2.1.5.1 Phylogenetic analysis and post translational modification

The predicted amino acid sequences of the mitochondrial proteins cytochrome b (*CytB*) and cytochrome c (*CytC*), together with the nuclear-encoded immune proteins NLRP3, IL-18, and STING, were subjected to comprehensive in silico protein sequence analysis. Multiple sequence alignments were performed using the MAFFT algorithm (29) to compare the predicted protein sequences with their orthologues from other species and to evaluate sequence conservation and evolutionary relationships. Signal peptide prediction was carried out using SignalP 3.0 (Technical University of Denmark) to determine the presence and putative cleavage sites of N-terminal signal peptides. Signal peptides play a critical role in directing newly synthesized proteins to the secretory pathway or cellular membranes, where they are subsequently cleaved to generate mature functional proteins. The leucine content of each predicted protein was calculated from the amino acid sequence to facilitate the identification of leucine-rich regions that may contribute to protein function.

To further characterize the structural and functional features of the predicted proteins, sequence-based analyses were performed using the ExPASy bioinformatics platform. Conserved functional motifs, including leucine-rich repeats (LRRs), leucine zipper motifs, leucine-rich nuclear export signals (NESs), glycosylphosphatidylinositol (GPI)-anchor attachment sites, and N-linked glycosylation sites, were identified. LRR domains, which are characteristic of numerous pattern recognition receptors, are essential for ligand recognition and pathogen sensing. Leucine zipper motifs were analyzed because of their role in mediating protein dimerization and protein–protein interactions. N-linked glycosylation sites were evaluated to assess their potential contribution to protein folding, stability, trafficking, and membrane localization, whereas NES motifs were identified to predict nucleocytoplasmic transport. GPI-anchor prediction was performed to identify proteins that may be tethered to the plasma membrane through post-translational lipid modification. In addition, Prosite was used to identify conserved LRR motifs and other functionally relevant protein signatures. Disulfide bond-forming cysteine residues were also predicted, as disulfide bonds play a critical role in protein folding, structural stability, and the maintenance of biologically active three-dimensional conformations.

Leucine-rich nuclear export signals (NESs) were predicted using the NetNES 1.1 Server (Technical University of Denmark). Potential O-linked glycosylation sites were identified using the NetOGlyc 3.1 server, whereas N-linked glycosylation sites were predicted using the NetNGlyc 1.0 server. Leucine zipper motifs were identified using the ExPASy bioinformatics tools (30). Secondary structural elements, including α-helices and β-sheets, were predicted using the NetSurfP server (Technical University of Denmark) (23), while putative domain linker regions were identified as described previously (31). Putative lipopolysaccharide (LPS)-binding (32) and LPS-signaling (33) sites were predicted through comparative sequence analysis based on homology with corresponding proteins from other species. These conserved functional regions are considered important for pathogen recognition, ligand binding, and the initiation of innate immune signaling pathways.

##### 2.1.5.2 Three-dimensional structure prediction and model quality assessment

Three-dimensional (3D) structural models of the mitochondrial proteins cytochrome b (*CytB*) and cytochrome c (*CytC*), together with the nuclear-encoded proteins NLRP3, IL-18, and STING, were generated using the SWISS-MODEL repository (34). Suitable structural templates exhibiting the highest sequence identity with the target proteins were identified using Position-Specific Iterated BLAST (PSI-BLAST). In addition, homology modeling was performed using the PHYRE2 server, which predicts protein tertiary structures based on template-assisted comparative modeling (35).

The predicted 3D models were visualized and analyzed using the PyMOL molecular graphics system. The stereochemical quality of each model was initially evaluated using the Structural Analysis and Verification Server (SAVES), followed by structural refinement and validation using the ProSA web server (36). Model quality was assessed based on stereochemical parameters and overall structural reliability. Furthermore, the NetSurfP server (23) was employed to predict relative surface accessibility, solvent-accessible surface area, secondary structural elements (α-helices, β-strands, and coils), and residue-specific structural propensity scores. Structural similarity between the predicted duck proteins and their corresponding template structures was evaluated by three-dimensional structural superimposition using TM-align (37). The degree of structural conservation was quantified by calculating the root mean square deviation (RMSD), which served as a measure of structural divergence between the aligned protein models.

##### 2.1.5.3 Protein-protein interaction network depiction

To investigate the potential protein–protein interaction network of the selected mitochondrial and nuclear proteins, interaction analyses were performed using the STRING database (version 9.1) (38). Functional associations among the proteins were evaluated based on STRING confidence scores, where interaction scores <0.3 were considered low confidence, scores between 0.3 and 0.7 indicated medium confidence, and scores >0.7 were regarded as high-confidence interactions. To further elucidate the biological functions and signaling pathways associated with these proteins, Kyoto Encyclopedia of Genes and Genomes (KEGG) pathway enrichment analysis was performed. The KEGG analysis identified the functional associations of the target proteins with related molecular components and provided insights into their potential roles in biological pathways involved in innate immunity, host defense, and cellular signaling.

##### 2.1.5.4 In silico study for the detection of the binding site of IL18 with *Pasteurella multocida*

*Molecular docking of host IL18 with the Pasteurella multocida surface proteins*.

Molecular docking analysis was performed to predict the potential binding interactions between host immune proteins and pathogen-derived molecules. Molecular docking is an in silico computational approach that predicts the binding orientation and affinity of a ligand toward the three-dimensional (3D) structure of a target protein. Docking simulations were carried out using the PatchDock algorithm, which identifies potential protein–ligand complexes based on geometric shape complementarity (39).

The surface antigen of *Pasteurella multocida* was used as the ligand, whereas the host immune protein IL-18 served as the receptor to investigate their potential molecular interactions. The resulting docking complexes were subsequently refined and re-scored using the FireDock server to improve docking accuracy and to identify the most energetically favorable binding conformations.

### 2.2 Diagnosis and confirmation of *Pasteurella multocida*

The ducks were initially diagnosed with *Pasteurella* infection based on characteristic clinical signs and preliminary symptoms. Subsequently, confirmatory diagnosis was established through molecular detection using *Pasteurella multocida*-specific primers in the infected samples.

Gross anatomical view of the liver in *Pasteurella* infected duck.

Different visceral organs, including liver from infected ducks were examined to support the clinical diagnosis of duck pasteurellosis.

#### 2.2.1 Confirmation through molecular detection.

In the next step, we follow the confirmation of the samples with a molecular PCR-based technique using the following primers as recommended by OIE, Paris. The primer used for molecular detection of *Pasteurella multocida* based on KMT1 gene is being listed in **Table 2**.

**Table 2:** Primers used for the molecular detection of *Pasteurella multocida*.

Target gene	Primer sequence	Product size
KMT1 (Partial)	F5′GCTGTAAACGAACTCGCCAC-3′ R5′ ATCCGCTATTTACCCAGTGG-3′	460bp

It involves a complex mechanism of various pathways, mitochondrial involvement through cytochrome B, cytochrome C, mitochondrial fisson, release of mitochondrial DNA and complex interaction with nuclear encoded proteins NLRP3, IL18 and STING.

## 3 Differential mRNA expression profiling for cytochrome B and cytochrome C, NLRP3, IL18 and sting in Bengal duck

### 3.1 Real time-polymerase chain reaction

#### 3.1.1 RNA estimation

The experimental procedures were performed as described in the earlier section on molecular characterization. Briefly, tissue samples were homogenized and immediately suspended in TRIzol reagent. Approximately 1 mL of homogenized tissue suspension was transferred into a 2 mL microcentrifuge (Eppendorf) tube, followed by the addition of 1 mL of chloroform. The mixture was thoroughly mixed and centrifuged at 10,000 rpm for 10 min at 4°C in a refrigerated centrifuge (Thermo Fisher Scientific).

Following centrifugation, three distinct phases were observed. The upper aqueous phase was carefully collected and transferred to a new microcentrifuge tube for RNA isolation. An equal volume of 100% isopropanol was added to the recovered aqueous phase, mixed gently, and incubated at room temperature (20–25°C) for 1 min, followed by centrifugation at 10,000 rpm for 10 min.

The resulting supernatant was discarded, and the RNA pellet was washed with an equal volume of 70% ethanol. The sample was again centrifuged at 10,000 rpm for 10 min, and the supernatant was carefully removed. The RNA pellet was then air-dried at room temperature and subsequently resuspended in nuclease-free water (Applied Biosystems, Cat. No. AM9930). The purified RNA was stored at −20°C for further use.

Qualitative analysis of total RNA.

The total RNA was quantified with NanoDrop-8000 (Thermo Scientific, Model No. 8000 Spectrophotometer), taking 1µl of each sample to determine the concentration and A260/280 ratio.

#### 3.1.2 First strand cDNA synthesis (reverse transcriptase -PCR)

Approximately 5 µL of total RNA was transferred into a PCR microcentrifuge tube, followed by the addition of 1 µL oligo(dT) primer, 1 µL 10 mM dNTP mix, and 13 µL nuclease-free distilled water. The reaction mixture was gently mixed and incubated at 65°C for 5 min to denature secondary RNA structures. The tubes were then rapidly chilled on ice for 5–7 min and briefly centrifuged.

Subsequently, 4 µL of 5× first-strand buffer and 2 µL of 100 mM dithiothreitol (DTT) were added to the reaction mixture, mixed thoroughly, and incubated at 37°C for 2 min. Thereafter, 1 µL of M-MLV reverse transcriptase (200 U/µL) was added, and first-strand cDNA synthesis was carried out at 37°C for 50 min, followed by enzyme inactivation at 70°C for 15 min. The entire reverse transcription protocol was performed in a thermal cycler (Applied Biosystems, Life Technologies) using a programmed cycling setup.

#### 3.1.3 Primer designing and synthesis

All the primers were designed using primer 3 software (v. 0. 4.0) as per the recommended criteria. Primers used in the experiment, were designed with DNASTAR and Primer 3software.

#### 3.1.4 Real-time PCR experiment (SYBR green based)

The primers were standardized with respective cDNA samples before being subjected to real-time PCR. The reactions were conducted in triplicate (as per MIQE Guidelines) with the total volume of the reaction mixture made up to 20µl. The experiment was conducted with Cytochrome B, cytochrome C, IL18, NLRP3, STING and GAPDH (housekeeping gene) primers calculated as per concentration. 1µl of cDNA was mixed with 10µl Hi-SYBr Master Mix (HIMEDIA MBT074) and rest volume made upto 20µl with nuclease-free water . Then the reaction was performed into the ABI 7500 system and run the reaction program. The delta-delta-Ct (ΔΔCt) method 2^- ΔΔCt^ was employed for the analysis of the result (40–42). The primers used for the reaction are being listed in **Table 3**, annealing temperature being set as 60C.

**Table 3:** Primers used for the differential mRNA expression profiling of immune response genes of duck.

Primer	Primer sequence
CYT B F	5′ ACCCTGACCCGATTCTTTC 3′
CYT B R	5′ GATGCGAGTTGCCCGATGA 3′
CYT C F	5′ TTCTCCAACCACAAAGACATTGGCAC 3′
CYT C R	5′ ACGTGGGAGATAATTCCAAATCCTG 3′
NLRP3 F	5′ ACAGCTTCACACACCTGCAC 3′
NLRP3 R	5′ GTGAAATTCTGCACCCGATT 3′
IL-18 F	5′ CTGATGACGATGAGCTGGAA 3′
IL-18 R	5′ CAAAAGCTGCCATGTTCAGA3′
STING F	5′ CGCAATGAGCCTGTAGGTTCC3′
STING R	5′ ACAAGCACAGCCTCTACGCAATC3′
GAPDH F	5′ ATGTTCGTGATGGGTGTGAA3′
GAPDH R	5′ CTGTCTTCGTGTGTGGCTGT3′

Statistical analysis.

Microsoft Excel was used for descriptive statistical analysis. Statistical analyses were performed using SYSTAT version 13.1 (SYSTAT Software Inc.). Gene expression levels were evaluated using the comparative Ct (ΔΔCt) method, and relative expression was calculated using the 2^-ΔΔCt^ approach (40–42).

## 4 Result

### 4.1 Characterization of mitochondrial and nuclear genes and derived peptide

#### 4.1.1 Characterization of the mitochondrial origin- cytochrome B derived peptide from duck liver

We sequenced the cytochrome b (*CYTB*) gene from Bengal duck and predicted its corresponding amino acid sequence (QVX28267.1) using in silico approaches. Subsequently, a three-dimensional (3D) structural model of cytochrome b was generated (**Figure 1a**).

**Figure 1:**
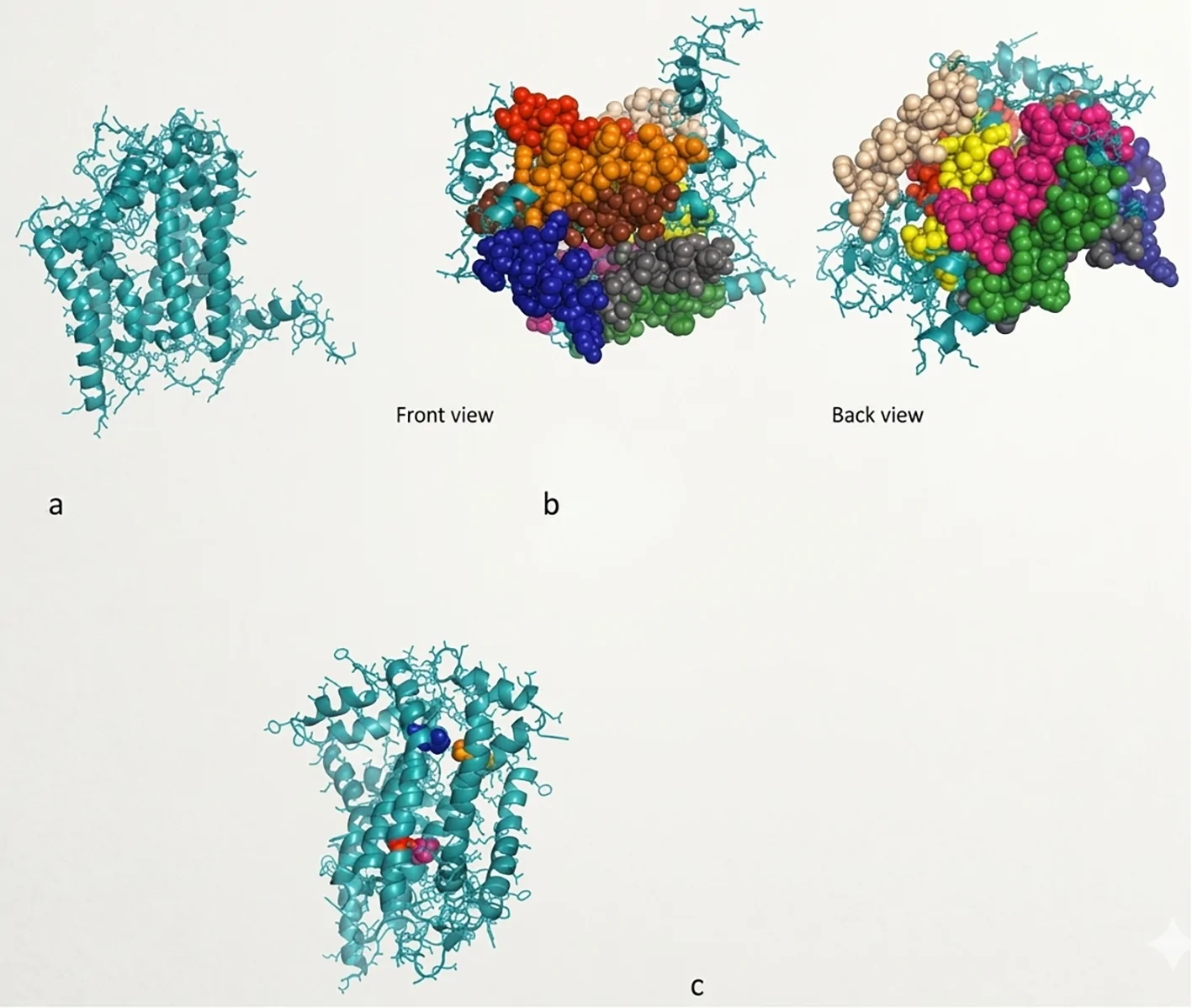
**(a)** 3D model for duck cytochrome B. **(b)** Important binding sites (haem and iron binding site) and topology for duck cytochrome B Red sphere: amino acid 34-54, heme binding site, transmembrane site, helical topology. Yellow sphere: Amino acid 84, axial binding residue, ligand, Fe (Iron binding site). Purple sphere: Amino acid(aa) 87-107, heme binding site, transmembrane site, helical topology. Chocolate sphere: aa 116-136, transmembrane site, helical topology. Blue: 151-171, transmembrane site, helical topology. Orange 179-199, transmembrane site, helical topology and heme binding site. Wheat 225-245, transmembrane site, helical topology. Gray 289-309 transmembrane site, helical topology. Hot pink 324-344 transmembrane site, helical topology. Forest green 350-370 transmembrane site, helical topology. **(c)** Other important binding sites for cytochrome B. Orange sphere: aa84 iron binding, axial binding residue, Warm pink sphere: aa 98 iron binding, axial binding residue, Blue sphere: aa 183, iron binding, axial binding residue, heme binding red sphere: aa 197 iron binding, axial binding residue.

In the next step, potential ligand-binding sites were predicted for iron and haem. The predicted iron-binding residues were located at amino acid positions 84, 98, 183, and 197. The haem-binding regions were identified within the following residue ranges: 34–54, 87–107, and 179–199, with an additional contributing residue at position 183. These predicted binding sites are illustrated in detail in **Figures 1b, c**, where they are highlighted using colored spheres for clear structural representation.

#### 4.1.2 Characterization of nuclear genes involved in mitochondrial function- cytochrome C derived peptide from duck liver

Cytochrome C for Bengal duck was sequenced and submitted to gene bank with accession number Bankit number 2970653. 3D structure was predicted (**Figure 2a**) with binding sites for iron and haem C (**Figure 2b**) was predicted and represented with respective colored spheres. Cytochrome C protein is encoded by nuclear genes, whereas, CytC protein functions in mitochondria.

**Figure 2:**
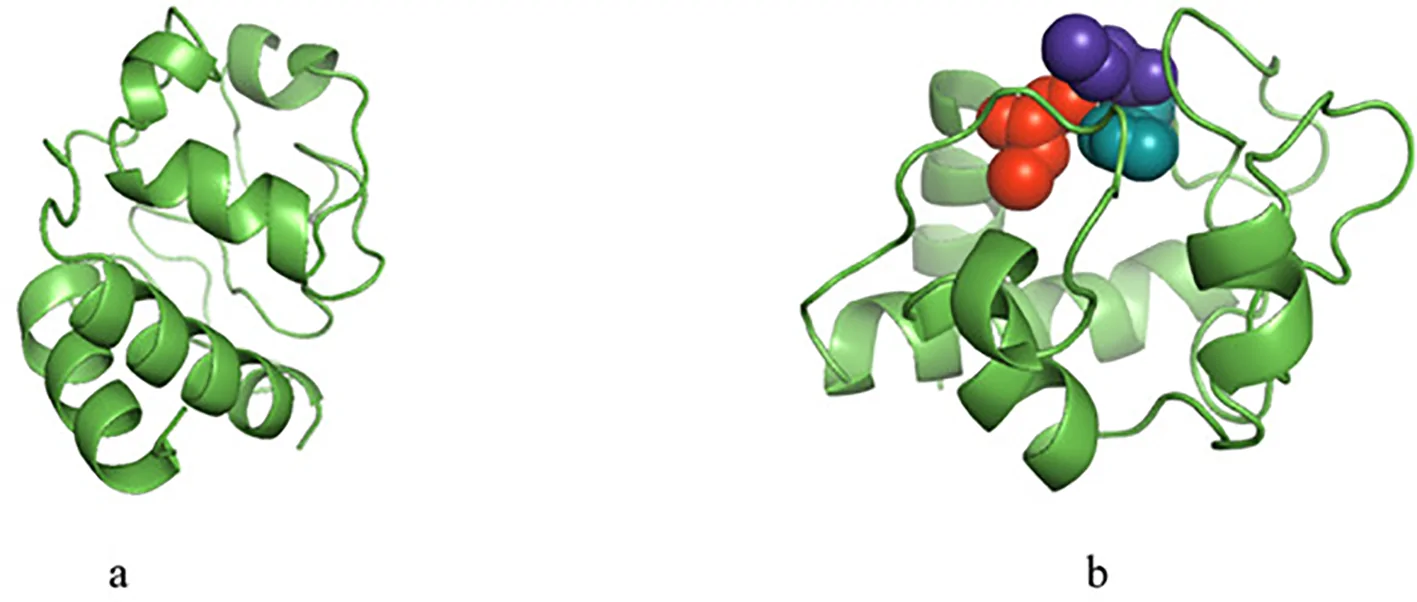
**(a)** 3D model for duck cytochrome C. **(b)** Important binding site for cytochrome C for duck. Red sphere: Amino acid 15: Heme C binding site, Blue sphere: amino acid 18: Heme C binding site, deep teal sphere: Iron binding site: axial binding residue.

#### 4.1.3 Characterization of nuclear gene

We have sequenced the genes from duck origin and submitted to gene bank with accession number for IL18 and Sting as bankit id 2970661 and bankit id 2970864 respectively. We have obtained the 3D structure for NLRP3 (**Figure 3**), IL18 (**Figure 4**), and Sting (**Figure 5**) gene of Bengal Duck with their respective binding sites.

**Figure 3:**
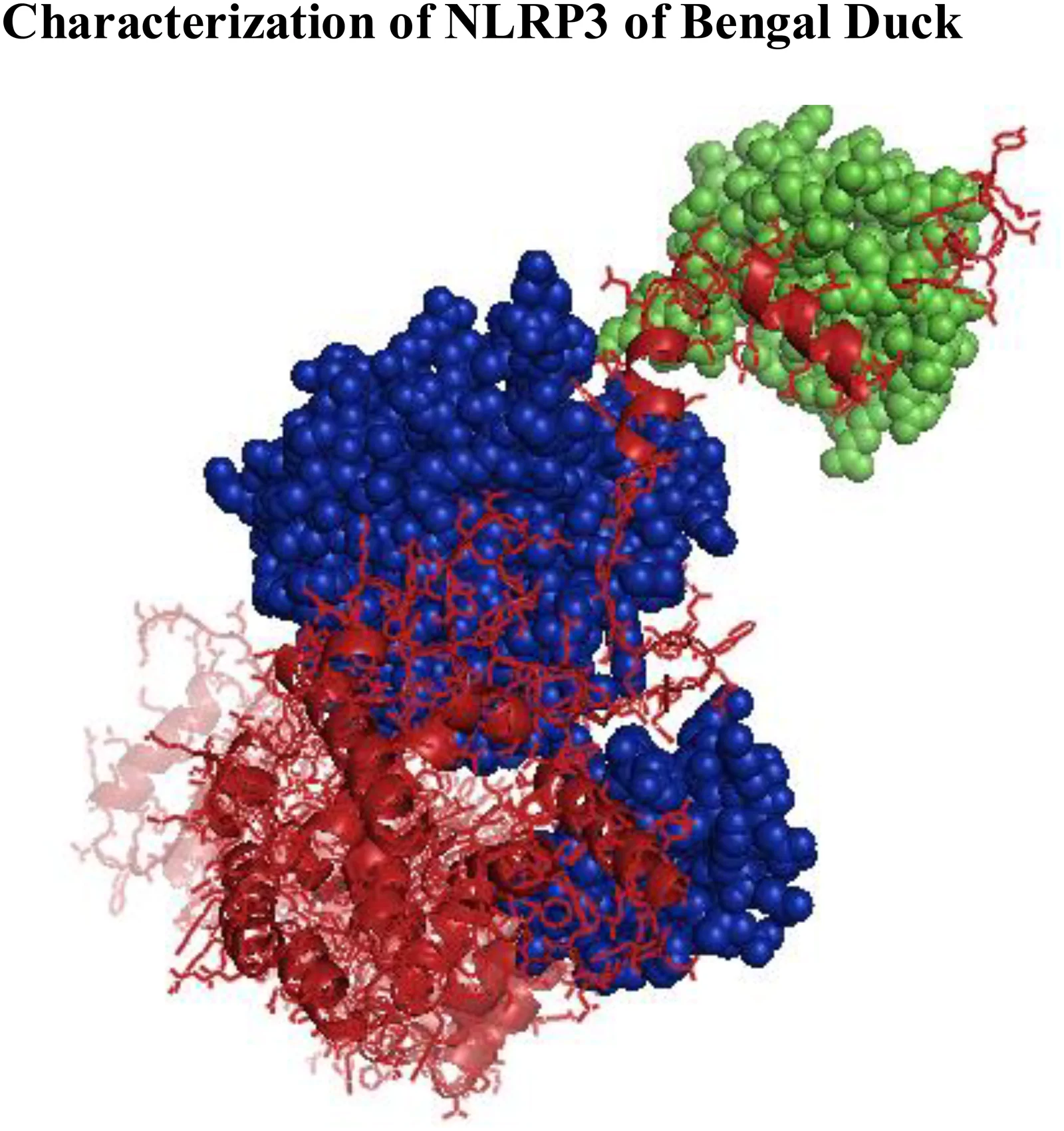
3D structure of NLRP3. Green sphere: 1-94: DAPIN or Pyrin domain. Blue sphere: 177-380: NACHT domain.

**Figure 4:**
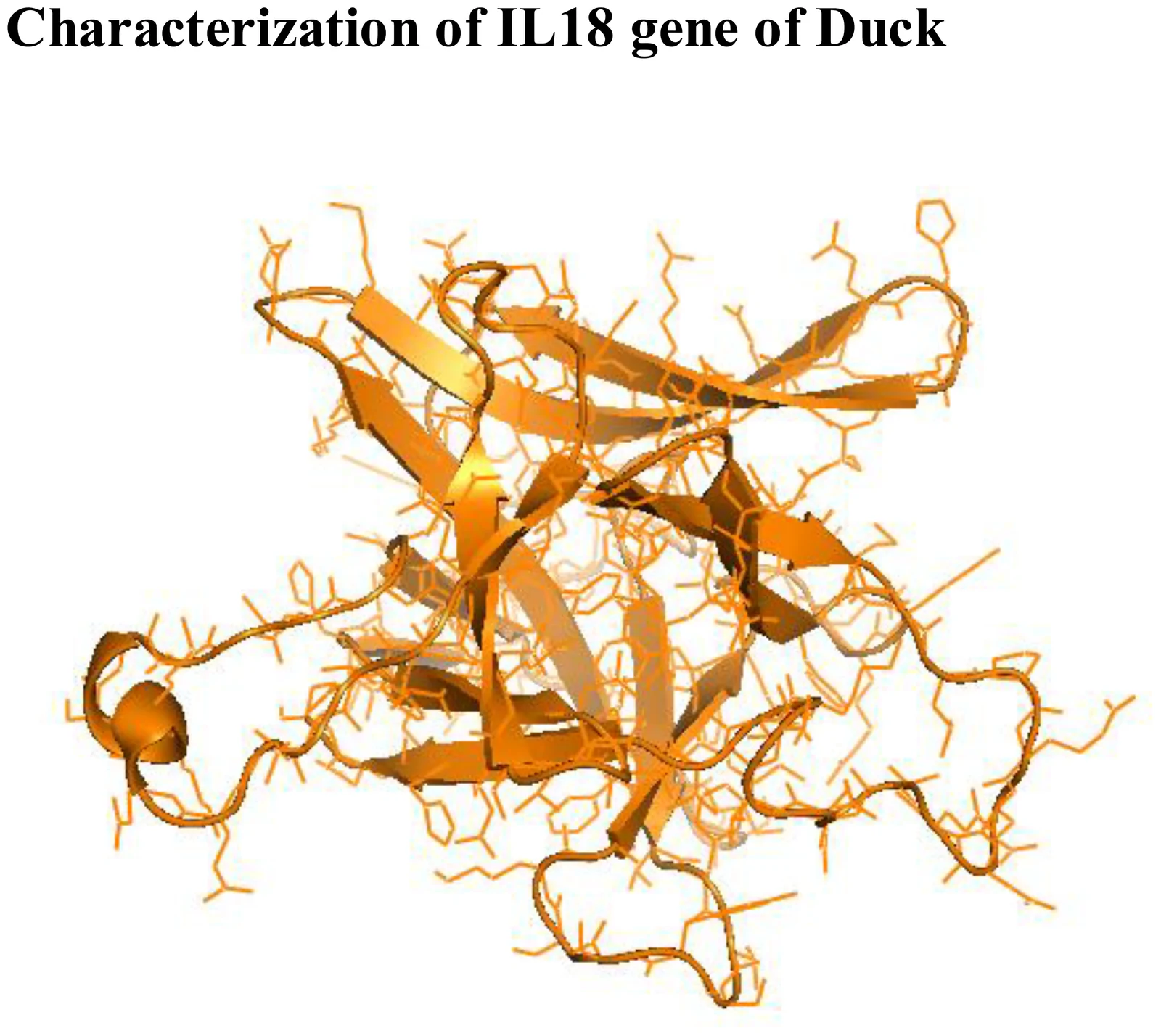
3D structure for IL18 for duck.

**Figure 5:**
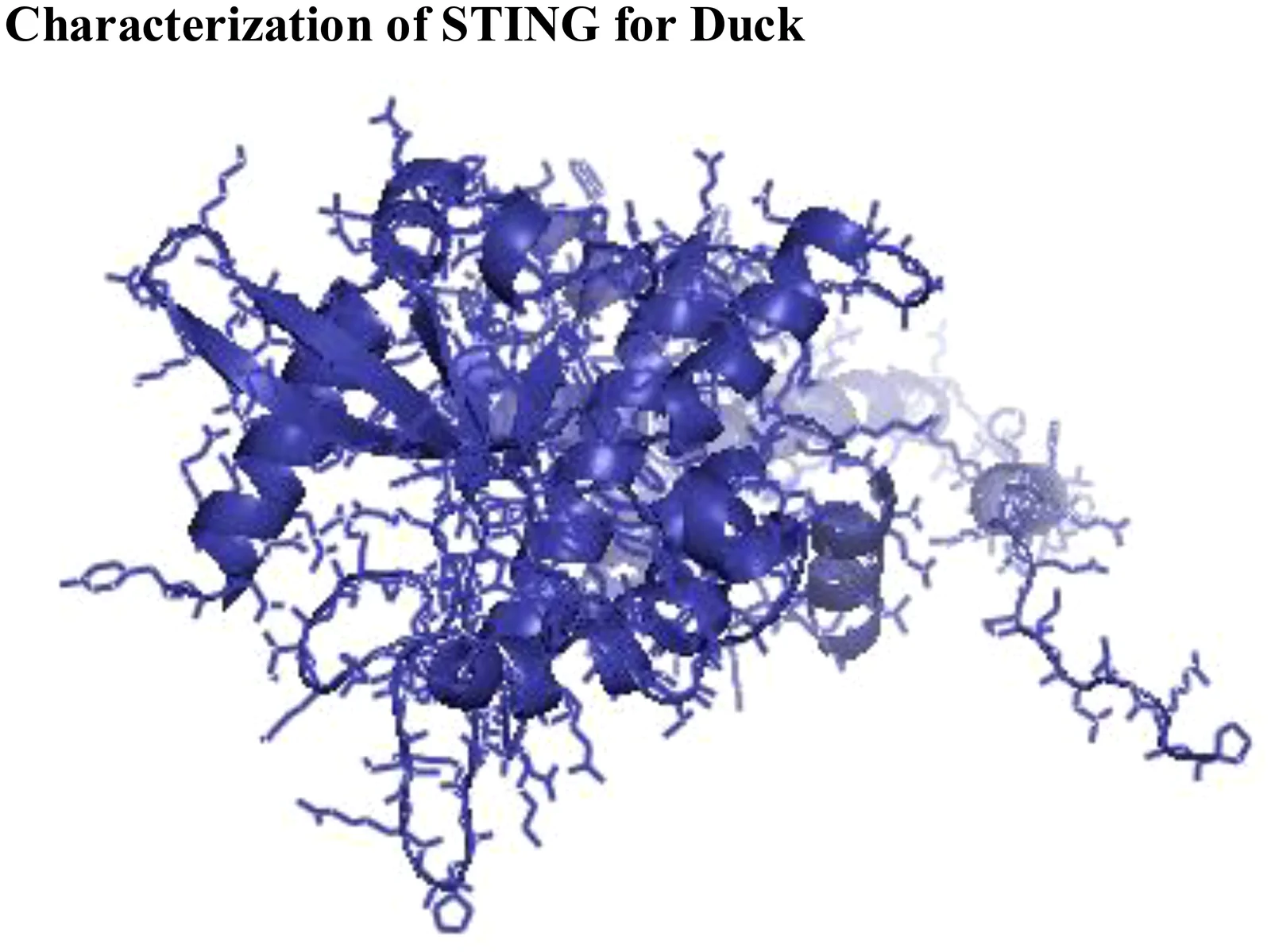
3D structure for STING for duck.

Functional domains identified for the Domain in Apoptosis and Interferon response (DAPIN) is an 80-100 residue domain found at the N-terminus of various vertebrate-specific viral proteins involved in apoptosis, cancer, inflammation and immune response. The DAPIN domain can be found alone or in association with other domains such as CARD, LRR, SPRY, Caspase or zinc finger B-box. It has been predicted that the DAPIN domain could have adapter function, coupling apoptosis and immune disorders. The DAPIN domain has been shown to be a protein-protein interaction domain capable of binding to other DAPIN domains. Secondary structure predicts that the DAPIN domain as predominantly α-helical, and it was proposed that it might belong to a superfamily of composite DEATH domains that includes a CARD, a DEATH domain (DD), and a DEATH effector domain (DED).The DAPIN domain has also been called pyrin domain, pyrin N-terminal homology domain (PYD) or PAAD (after pyrin, AIM, ASC death-domain-like protein families).

A protein domain that has been conserved throughout evolution is the NACHT domain. Proteins involved in MHC transcription and apoptosis both have this NTPase domain. First, the names of some of the proteins that contain it are NAIP (NLP family inhibitor of apoptosis protein), CIITA (also known as C2TA or MHC class II transcriptional activator), HET-E (Podospora anserina incompatibility locus protein), and TEP1 (also known as TP1 or telomerase-associated protein).

The NACHT domain consists of 300 to 400 amino acids. This is a predicted nucleoside triphosphatase (NTPase) domain available in animal, fungal and bacterial proteins. It is observed in association with other domains such as a CARD domain (InterPro: IPR001315), a pyrin domain (InterPro: IPR004020), a HEAT repeat domain (InterPro: IPR004155), a WD40 repeat (InterPro: IPR001680), a leucine-rich repeat (LRR) or BIR repeats (InterPro: IPR001370).

The NACHT domain comprises of seven distinct conserved motifs, including an ATP/GTPase-specific P-loop, an Mg^2+^ -binding site (Walker motifs A and B, respectively), along five more specific motifs. Unique features of the NACHT domain comprises of the prevalence of “minor” residues (glycine, alanine, or serine) directly C-terminal to the Mg^2+^ -coordinating aspartate in the Walker B motif, in place of the second acidic residue prevalent in other NTPases. The second acidic residue is detected in NACHT-containing proteins two positions downstream. Distal motif VII contains a conserved pattern of polar, aromatic, and hydrophobic residues unique in NTPase family.

### 4.2 Differential mRNA expression profiling for mitochondrial genes

Differential mRNA expression profiling for cytochrome B and cytochrome C have been depicted in **Figures 6a, b** respectively. Gene expression of both Cytochrome B and cytochrome C was observed to be better in healthy ducks in comparison to infected ducks.

**Figure 6:**
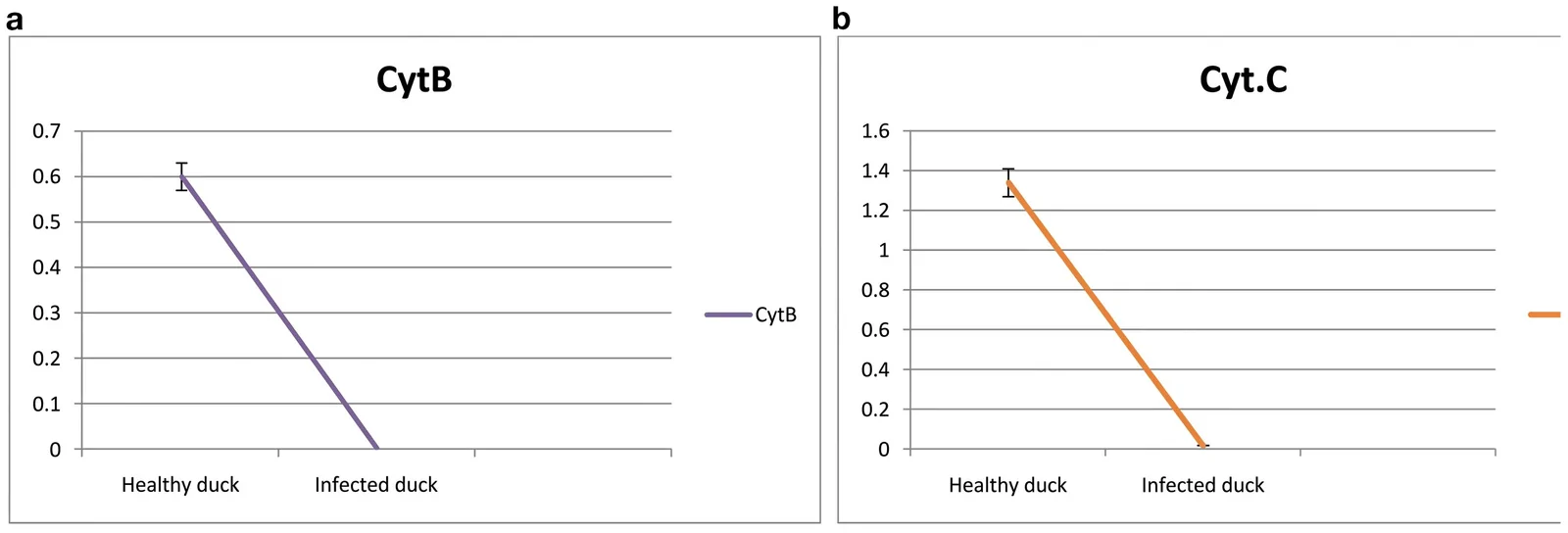
**(a)** Differential mRNA expression profiling for Cytochrome B gene in duck with respect to *Pasteurella multocida* infection. **(b)** Differential mRNA expression profiling for Cytochrome C gene in Duck with respect to *Pasteurella multocida* infection.

Differential mRNA expression profiling for nuclear (NLRP3, IL18 and STING) genes.

Differential mRNA expression profiling for NLRP3 (**Figure 7a**), IL18 (**Figure 7b**) and STING (**Figure 7c**) gene in duck liver tissue reveal better expression of these genes in helathynducks in comparison to infected ducks.

**Figure 7:**
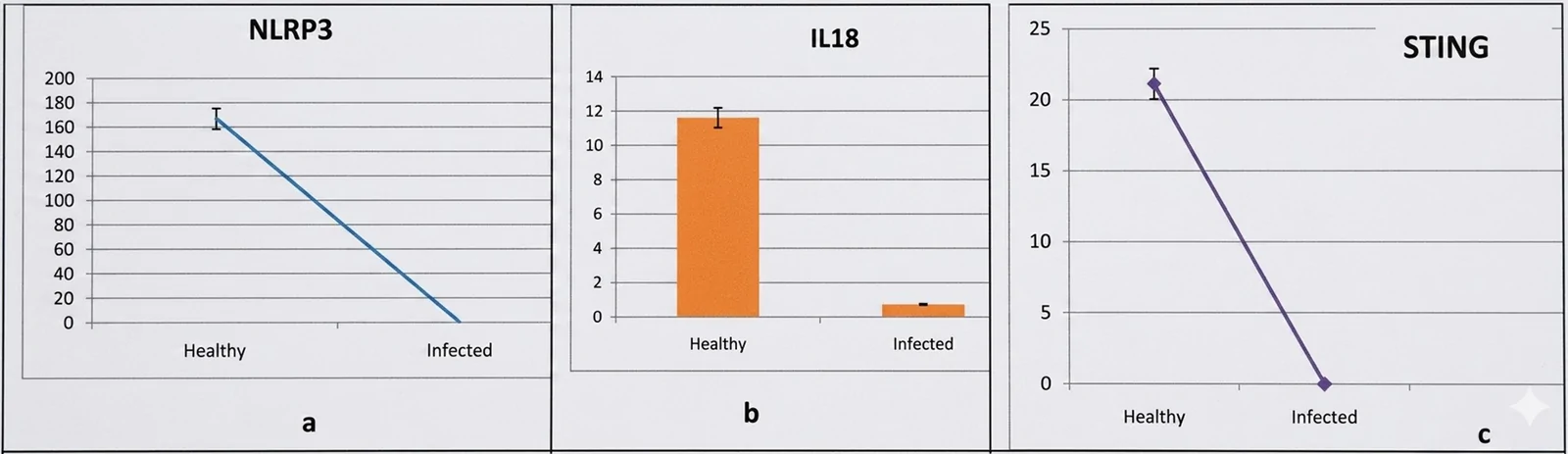
**(a)** Differential mRNA expression profiling for NLRP3 gene in duck. **(b)** Differential mRNA expression profiling for IL18 gene in duck. **(c)** Differential mRNA expression profiling for Sting gene w.r.t Pasteurellosis in liver tissue.

Molecular docking of IL18 with *Pasteurella multocida*.

Molecular docking analysis was conducted for IL18 with certain surface protein of P*asteurella multocida* predicted to bind with membrane protein, lipoprotein, zinc transporter membrane, electron transporter RnfD, cell division protein FtsW.

Patchdock score was observed to be high (21096) for IL18 with *Pasteurella multocida* membrane protein. On the otherhand patchdock score was less for lipoprotein 14344. And zinc transporter membrane as 14598, lipopolysaccharide16374, *Pasteurella_multocida* electron_transporter_RnfD 19176, cell division protein FtsW 16392.

Molecular docking for IL18 with the duck *Pasteurella multocida* membrane protein indicates a good score and is represented in **Figure 8a**. (IL18: Red for IL18, blue for membrane protein), whereas **Figure 8b** indicates the binding sites for IL18 with the *Pasteurella multocida* membrane protein ranges from Ala31 to Glu196.

**Figure 8:**
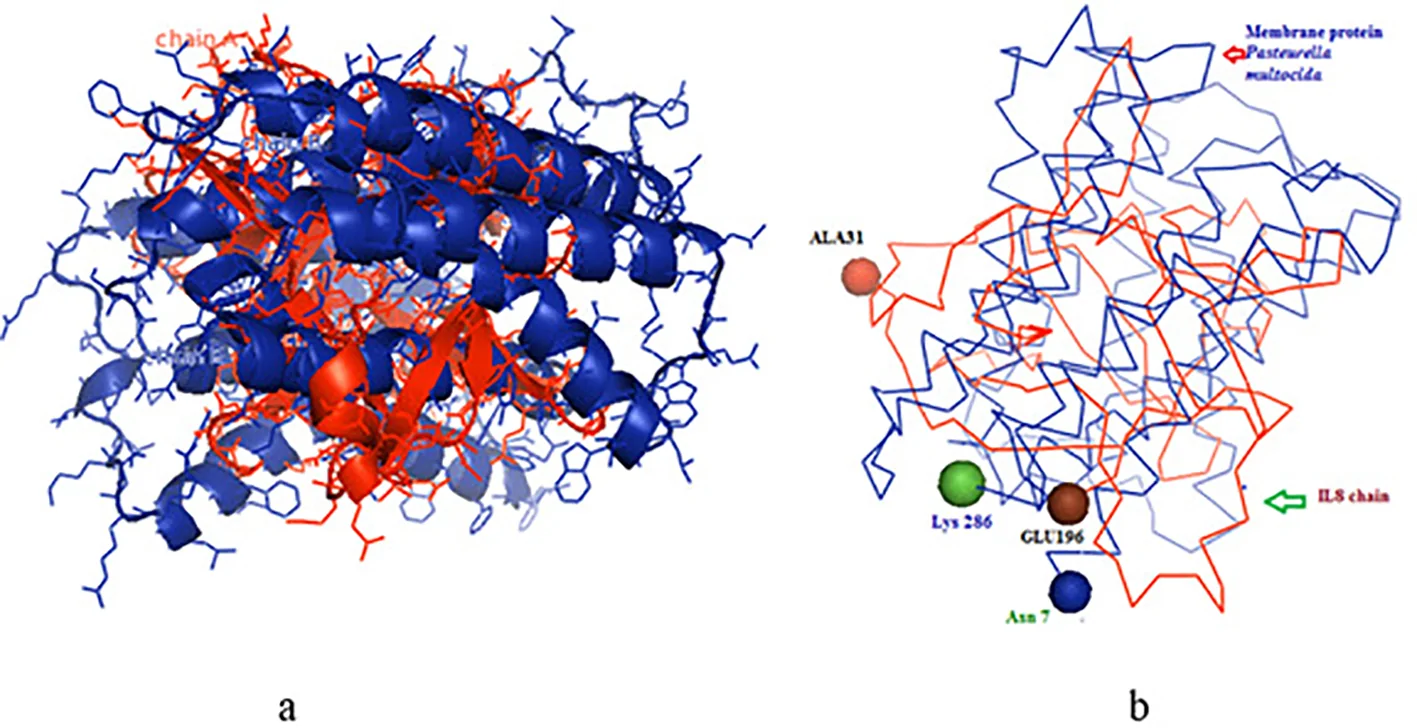
**(a)** Molecular docking for duck IL18 with the *Pasteurella multocida* membrane protein. **(b)** Binding sites for IL18 with membrane protein for *Pasteurella multocida*.

#### 4.2.1 Interaction––––string analysis

In any biological process, the proteins act together and interact with each other. The molecular interaction of IL18, NLRP3 and STING protein have been depicted in **Figures 9a–c**.

**Figure 9:**
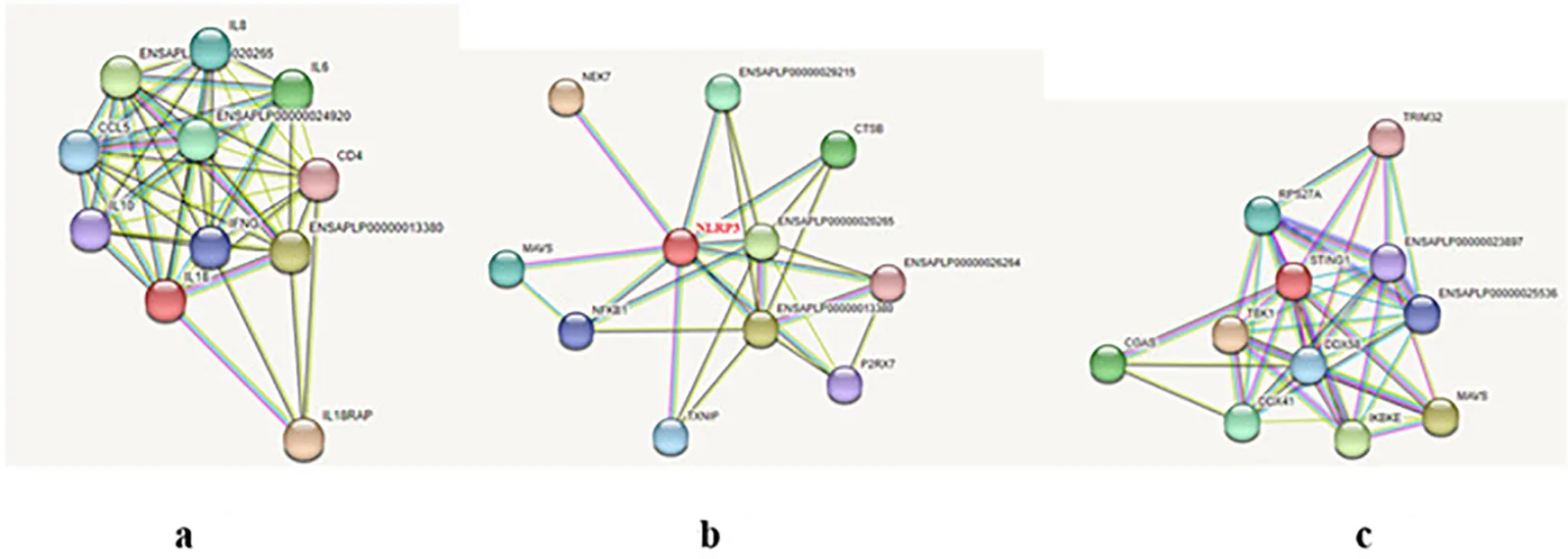
**(a)** Molecular interaction analysis for IL18 with other genes. **(b)** Molecular interaction analysis for NLRP3 for duck with other genes. **(c)** Molecular interaction analysis for STING for duck with other genes.

Strong association of the proteins (NLRP3, STING) was observed with the mitochondrial protein MAVS (Mitochondrial antiviral signaling) protein. MAVS is an essential component of the innate immune system, primarily located on the outer membrane of mitochondria. It acts as a signaling hub that is activated upon the detection of viral RNA, leading to the production of antiviral cytokines and interferons, which are critical for mounting an effective immune response against viral infections.

##### 4.2.1.1 Kegg analysis

The biological pathway analysis for IL10, NLRP3, STING gene and complex interaction of mitochondria and nuclear genes are being depicted in **Figures 10**–**13**. Innate immune responses are being governed by various pathways, namely, cytosolic DNA sensing pathway, NOD like receptor signaling pathway, pathway for immune response against Salmonella infection, necropsis pathway. In the above pathways, it is evident that the immune response is being governed by various pathways. The notable amongst them are mentioned here. The pathways involve the important role of mitochondria as well as immune response genes of nuclear origin as discussed in current context. Pathway for immune response against Salmonella infection involves mitochondria, including mitochondrial protein MAVS as well as NLRP3, IL18 molecules (**Figure 10**). Cytosolic DNA sensing pathway leading to antibacterial immunity involves a co-ordinated mechanism of mitochondria as well as NLRP3, STING and IL18 (**Figure 11**). NOD like receptor signaling pathway involves mitochondria, including mitochondrial protein MAVS as well as NLRP3, STING molecules (**Figure 12**). Necropsis pathway involves mitochondria, including mitochondrial protein MAVS as well as NLRP3 molecules (**Figure 13**).

**Figure 10:**
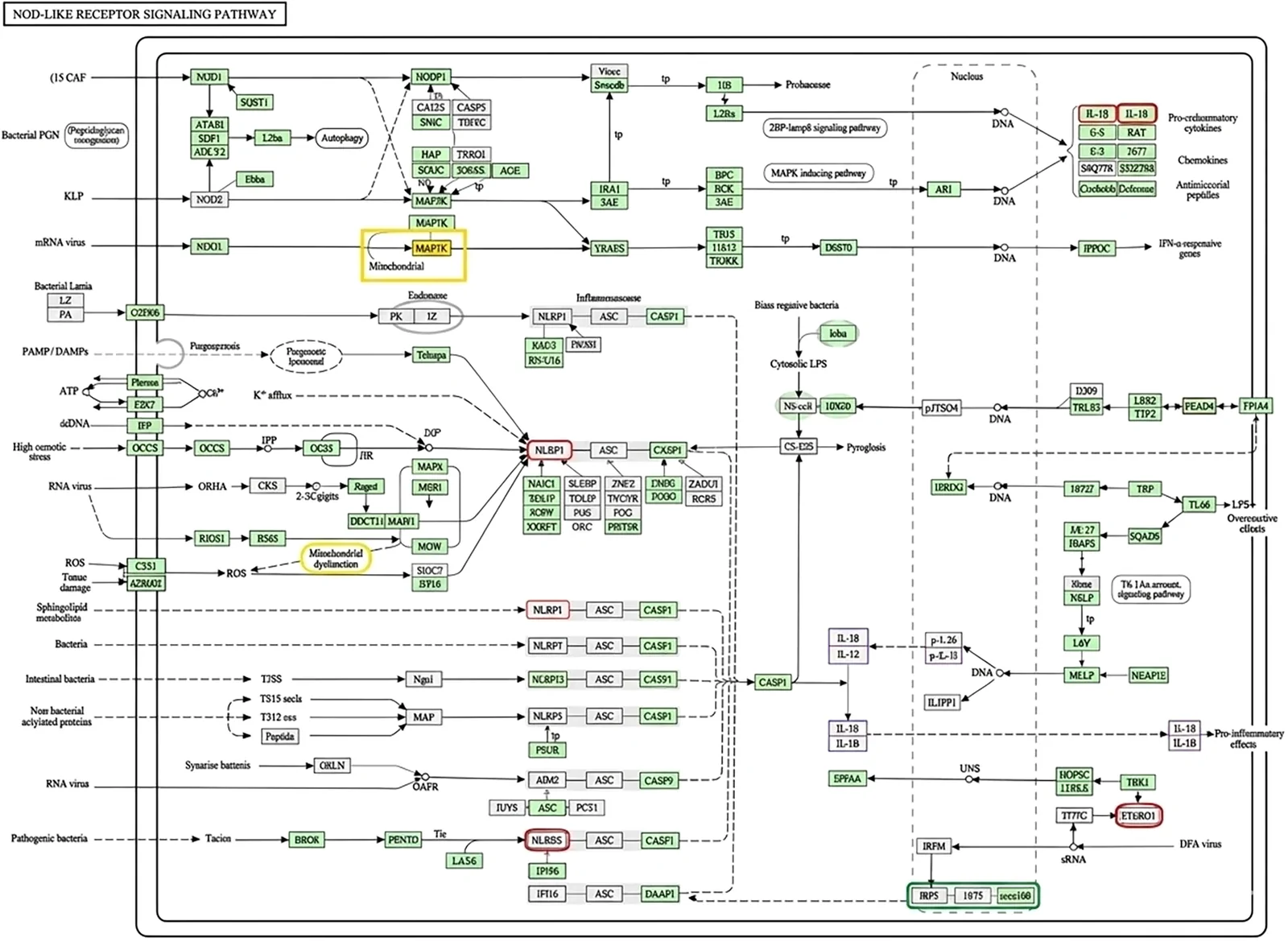
Molecular pathway analysis for IL18/NLRP3/STING gene for antibacterial activity in duck.

**Figure 11:**
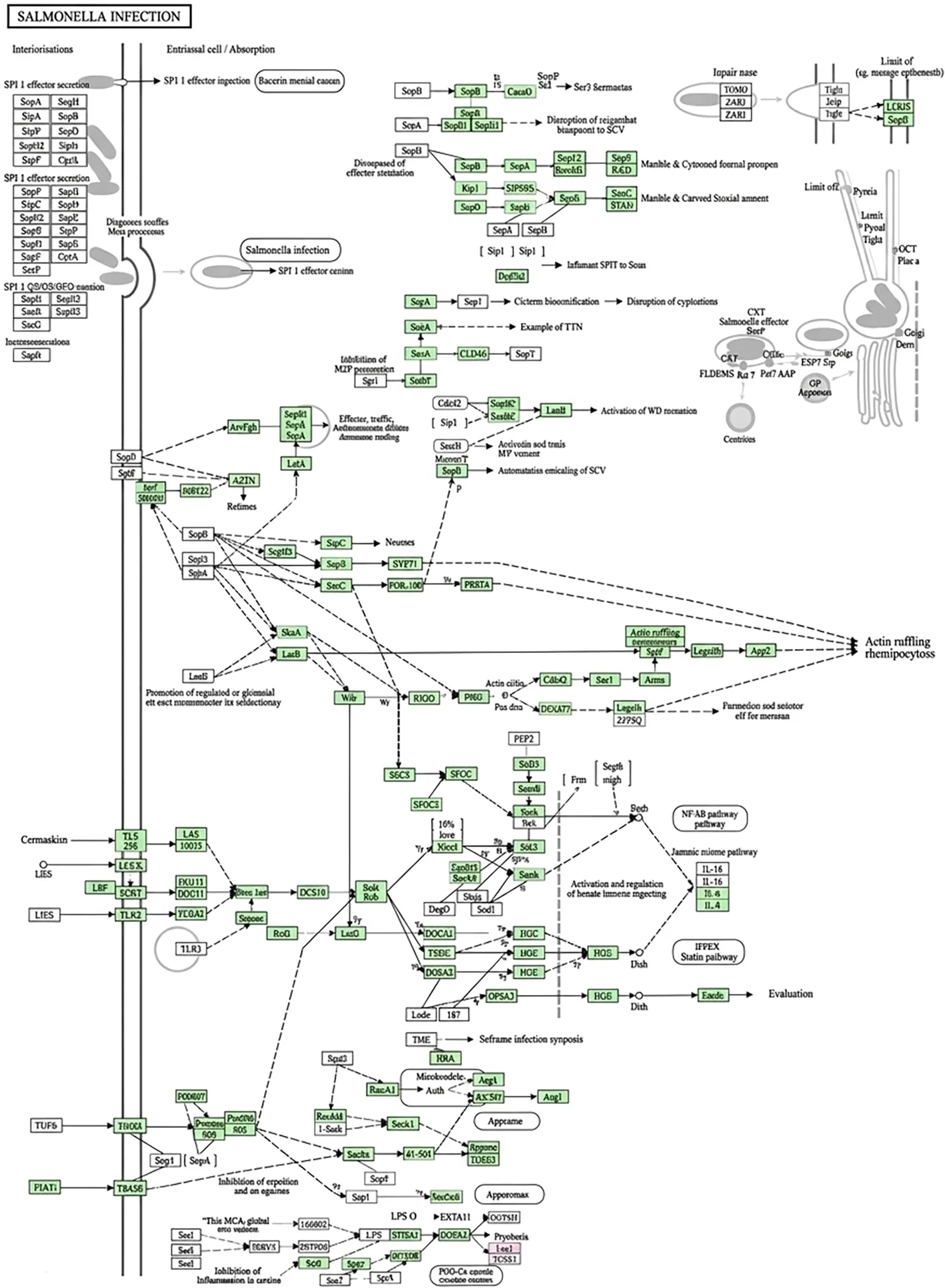
Molecular pathway analysis for IL18, STING and NLRP3 gene in duck.

**Figure 12:**
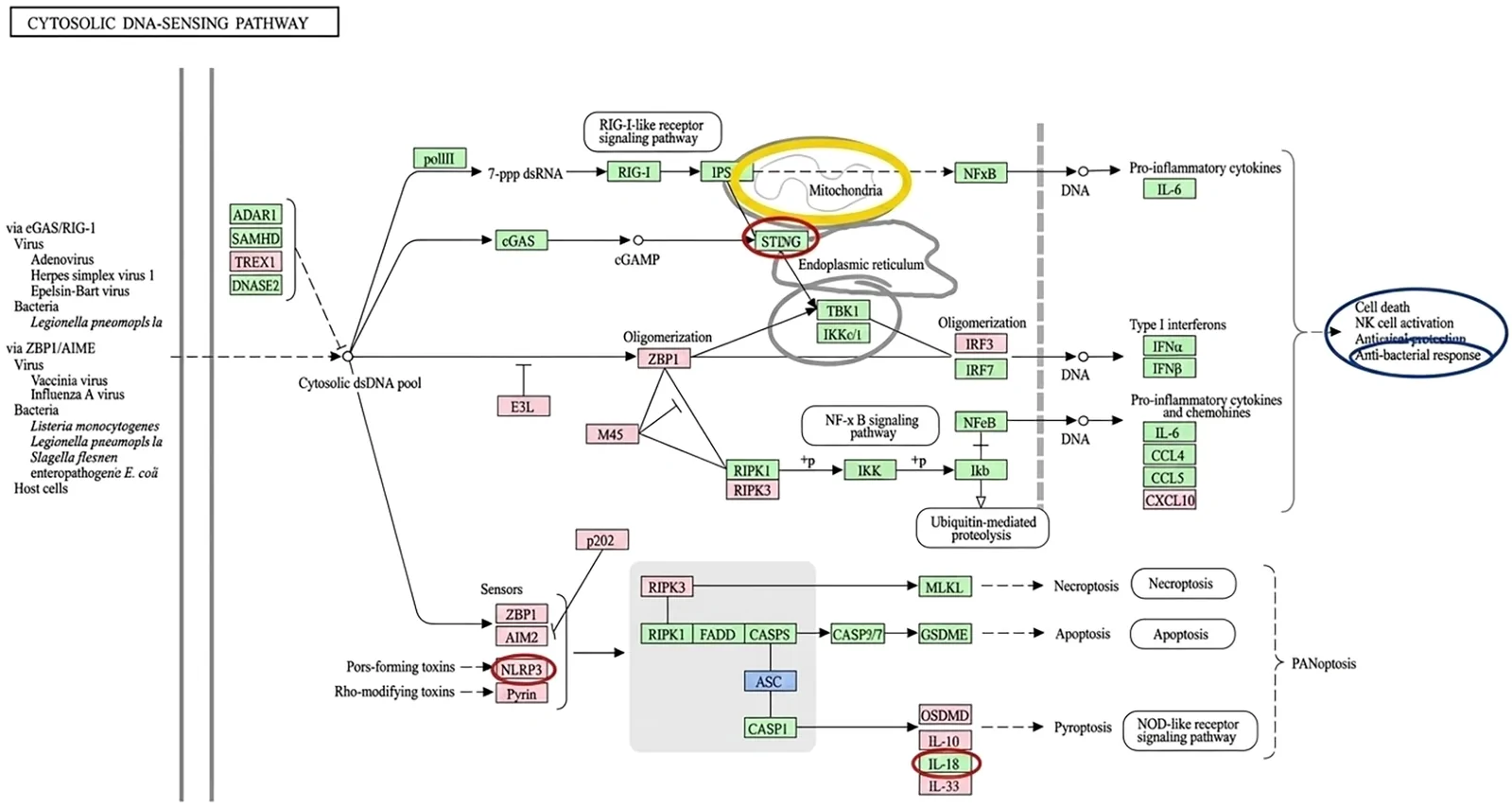
Molecular pathway analysis for IL18 gene in duck in response to bacterial infection.

**Figure 13:**
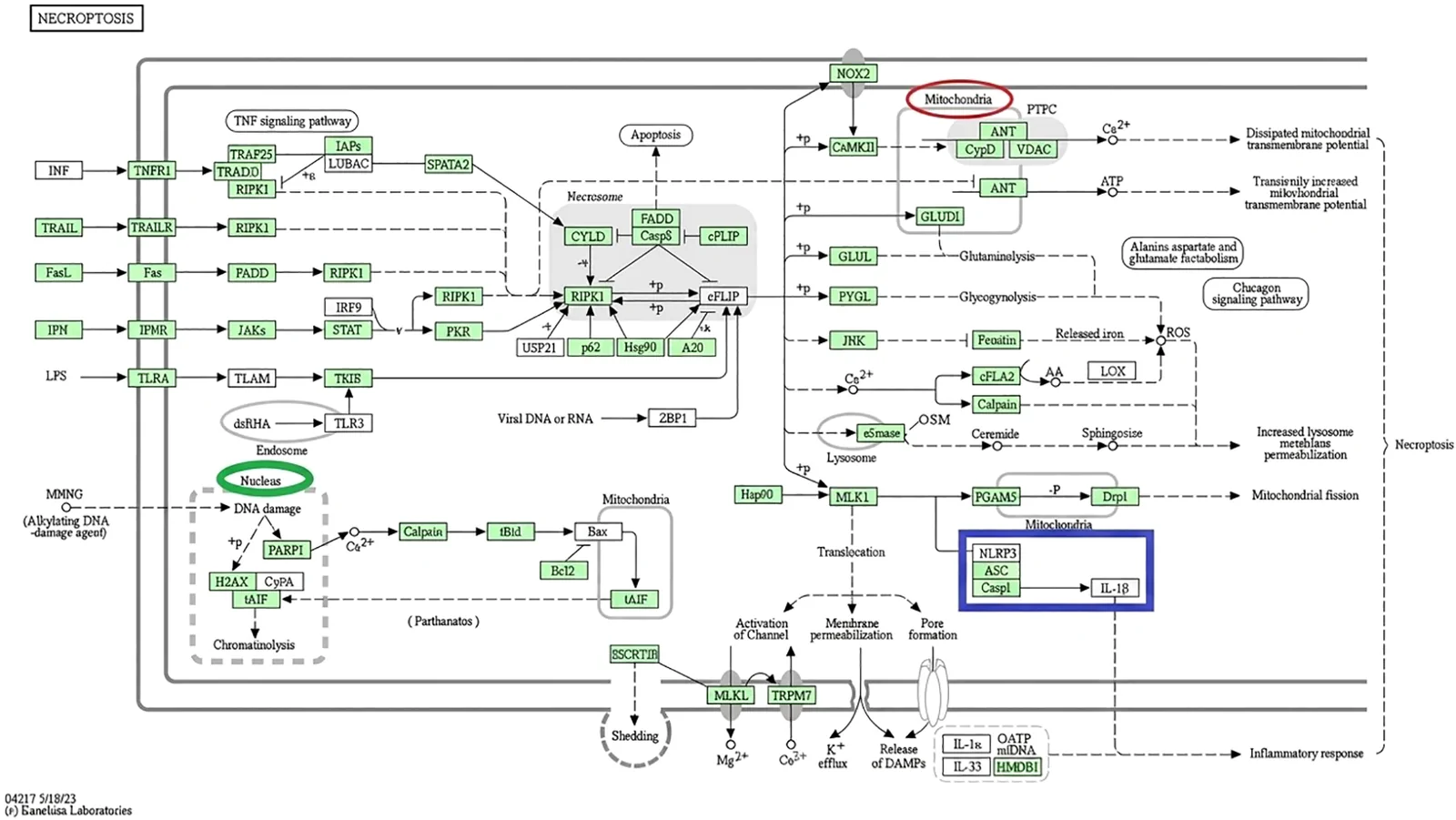
Molecular pathway analysis for NLRP3 gene and interaction of mitochondria DNA with nuclear genes in duck.

The schematic representation for the role of mitochondrial as well as nuclear immune response molecule acting synergistically for antibacterial immunity is represented in **Figure 14**. It involves a complex mechanism of various pathways, mitochondrial involvement through cytochrome B, cytochrome C, mitochondrial fisson, release of mitochondrial DNA and complex interaction with nuclear encoded proteins NLRP3, IL18 and STING.

**Figure 14:**
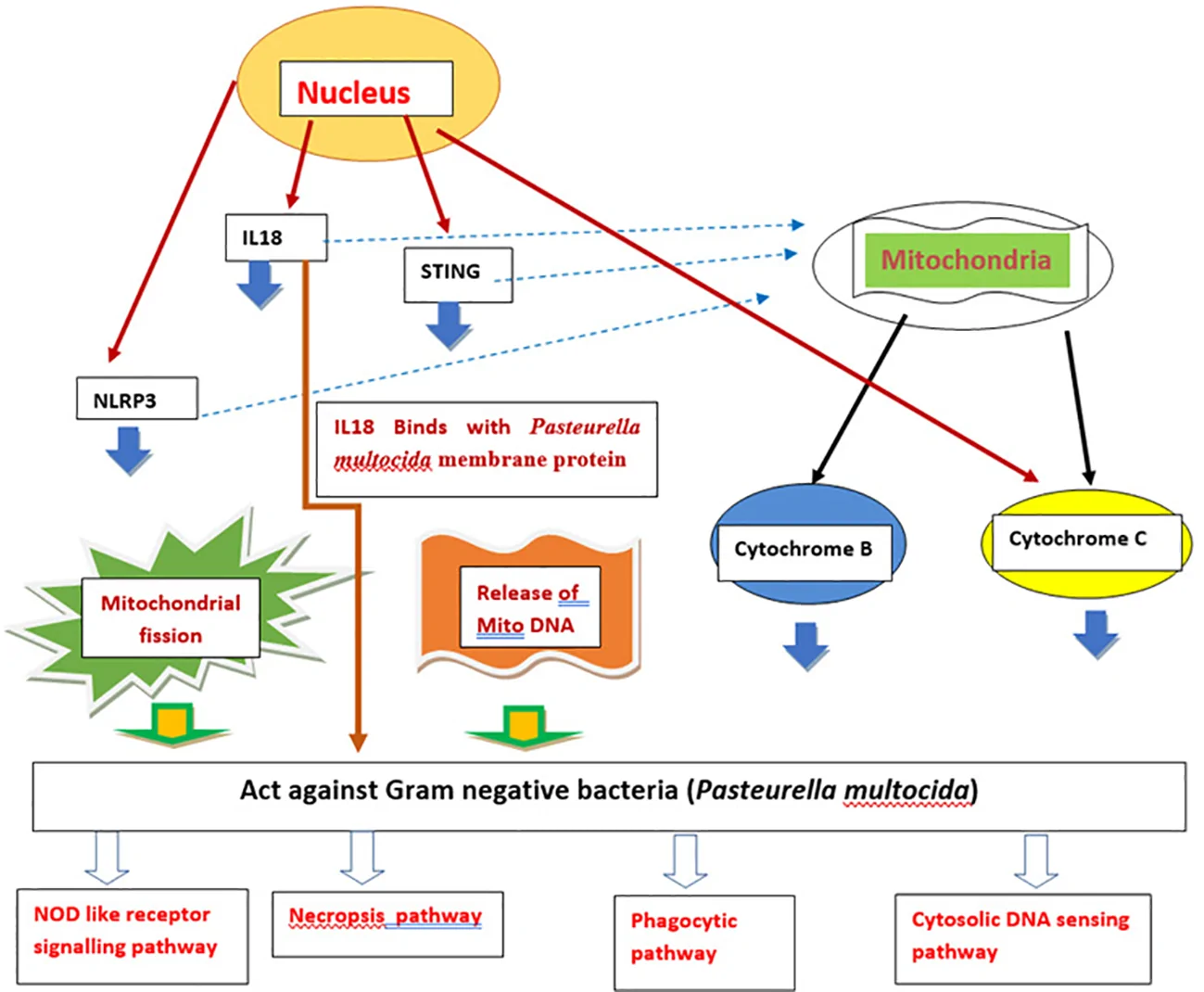
Schematic representation for the role of mitochondrial as well as nuclear immune response molecule synergistically acting for antibacterial immunity.

## 5 Discussion

In the present study, we investigated the molecular mechanisms underlying host resistance to duck pasteurellosis using a duck model, with particular emphasis on the coordinated interplay between mitochondrial-encoded proteins and nuclear-encoded immune response molecules during bacterial infection. To the best of our knowledge, this work represents a novel contribution and the first report describing mitochondrial–nuclear crosstalk in the context of duck pasteurellosis.

We performed a comprehensive characterization of key mitochondrial and nuclear genes in indigenous Bengal ducks. Mitochondrial protein-coding genes, including cytochrome b, along with the nuclear gene encoding cytochrome c, were analyzed through sequence characterization, three-dimensional structural modeling, and identification of functionally important domains. In parallel, nuclear-encoded innate immune response genes, including *IL18*, *NLRP3*, and *STING*, were characterized using in silico structural modeling and domain-based functional annotation.

For functional assessment, experimental ducks were naturally exposed to *Pasteurella multocida*. Birds that remained clinically unaffected were classified as the healthy group and confirmed negative through molecular detection, whereas those that developed infection and subsequently succumbed to disease were categorized as the infected group that were further confirmed to be positive with molecular detection for *Pasteurella multocida*. Liver tissues collected from both groups were subjected to differential mRNA expression profiling. Higher expression levels of mitochondrial genes were observed in healthy individuals compared with infected birds. In particular, cytochrome b and cytochrome c exhibited significantly increased expression in healthy ducks, suggesting enhanced mitochondrial activity in hepatocytes. Better expression of cytochrome B is an indirect way of indication of more mitochondrial copy number. It is well known that mitochondria in a cell exists as multiple copy numbers, unlike nucleus. Pathway analyses further supported a potential role of mitochondria in antibacterial immune responses and host defense mechanisms.

Pathway analyses indicate that mitochondria play a central role in antibacterial innate immune responses, particularly through cytosolic DNA-sensing pathways. Mitochondrial signaling contributes to the activation of NF-κB, which in turn promotes the production of pro-inflammatory cytokines, including interleukin-6 (IL-6). In parallel, the stimulator of interferon genes (STING) pathway mediates antibacterial defense through activation of TBK1 and IKK, leading to phosphorylation and activation of IRF3 and IRF7, and subsequent induction of type I interferons (IFN-α and IFN-β). IL-18 exerts its antibacterial effects primarily by promoting inflammatory cell death, including pyroptosis, through NOD-like receptor (NLR) signaling pathways, whereas NLRP3 functions as a central inflammasome component that integrates multiple danger signals and can contribute to inflammasome-driven inflammatory cell death pathways. Mitochondria are actively involved in NLR-mediated signaling, and coordinated interactions among NLRP3, IL-18, and STING appear to be critical for mounting effective antibacterial immune responses. Pathogens may directly target mitochondrial function through MAVS-associated signaling pathways or indirectly induce mitochondrial dysfunction, resulting in excessive production of reactive oxygen species (ROS). Consistent with these observations, pathway analyses of infections caused by comparable Gram-negative bacteria, such as *Salmonella* spp., also highlight the involvement of mitochondrial signaling in conjunction with NLRP3 and IL-18-mediated immune responses. Furthermore, necroptosis-related pathway analyses suggest a functional interaction between mitochondrial dynamics and NLRP3 activation, potentially involving infection-induced mitochondrial fission.

In this study, higher expression of the mitochondrial gene cytochrome b (*CytB*) was observed in healthy ducks compared with infected individuals, suggesting enhanced mitochondrial activity in the former group. This observation is consistent with pathway analyses indicating that mitochondria play an important role in antibacterial defense against *Pasteurella* infection. Although increased cytochrome b expression may reflect improved mitochondrial function, it cannot be directly interpreted as evidence of increased mitochondrial copy number based on transcript abundance alone. In addition, cytochrome c (*CytC*), a key component of the mitochondrial electron transport chain located in the inner mitochondrial membrane, also showed higher expression in healthy ducks. Collectively, elevated expression of both *CytB* and *CytC* in healthy individuals suggests a potential association between mitochondrial functional status and host resistance to bacterial infection.

These mitochondrial components may contribute to antibacterial immune responses through coordinated involvement in NOD-like receptor (NLR) signaling, phagocytic pathways, and cytosolic DNA-sensing pathways, thereby supporting a role for mitochondria in innate immune defense during *Pasteurella* infection. Evidence from this study suggests that the detected transcripts may, at least in part, reflect cytoplasmic mRNA derived from mitochondrial DNA released or leaked into the cytosol. Differential expression analysis revealed significantly higher expression of mitochondrial genes cytochrome b (*CytB*) and cytochrome c (*CytC*) in healthy ducks compared with infected individuals. Similarly, nuclear-encoded immune response genes, including *IL18*, *NLRP3*, and *STING*, exhibited elevated expression in the healthy group. These findings indicate a positive association between mitochondrial gene expression and the expression of key nuclear immune response genes. KEGG pathway analysis further supports a model in which mitochondrial stress signals and/or mitochondrial DNA (mtDNA) release may contribute to the activation of multiple immune pathways, including the cytosolic DNA-sensing pathway, NOD-like receptor signaling, antibacterial immune response pathways, and necroptosis-related signaling. In addition, STRING network analysis revealed extensive protein–protein interaction networks among the analyzed immune-related genes, indicating a coordinated signaling cascade that may collectively contribute to antibacterial host defense.

Previous studies have described multiple mechanisms by which mitochondrial DNA (mtDNA) can be released during infection and subsequently modulate nuclear-encoded immune responses, ultimately contributing to antimicrobial defense mechanisms (21). Accordingly, ducks that survive natural exposure are expected to exhibit a more effective mtDNA-mediated immune activation, contributing to enhanced host resistance. Released mtDNA has been shown to activate the cyclic GMP–AMP synthase–stimulator of interferon genes (cGAS–STING) signaling pathway, a central axis in innate immune sensing of cytosolic DNA. In addition, mtDNA release has been extensively investigated in the context of regulated cell death (43, 44). West et al. further demonstrated that mitochondrial transcription factor A (TFAM) deficiency promotes mitochondrial stress and improper mtDNA packaging (45), resulting in its release into the cytoplasm, where it engages cGAS and triggers downstream type I interferon responses (46).

Earlier studies have highlighted the role of mitochondrial DNA (mtDNA) in the context of *Mycobacterium tuberculosis* infection. Following infection with *M. tuberculosis*, both mycobacterial DNA and host-derived mitochondrial stress responses have been implicated, leading to the release of mtDNA into the cytoplasm (47). This observation suggests that mitochondrial stress and dynamics are key contributors to infection-induced mtDNA release in *M. tuberculosis*–infected cells. Previous work has also reported cytochrome c release from mitochondria in *M. tuberculosis*–infected cells, indicating a potential role for BAX/BAK-dependent mitochondrial outer membrane permeabilization in infection-associated mtDNA release (48). A growing body of evidence further supports the involvement of mtDNA in regulating cell death pathways, including apoptosis, and its broader impact on immune modulation and disease resistance (49)– (50). Mitochondrial permeability has been shown to be influenced by NF-κB–dependent anti-tumor and stress response pathways under conditions of caspase deficiency (51). In addition, the role of cytochrome c in maintaining mitochondrial integrity and its association with disease processes has been widely documented (52)– (53). Nuclear DNA can also be released extracellularly through the formation of neutrophil extracellular traps (NETs), a highly regulated process involving chromatin decondensation and histone citrullination, accompanied by controlled plasma membrane permeabilization that ultimately leads to cell death (54). Furthermore, extensive cross-talk between mitochondrial pathways and inflammasome components such as NLRP3 (55, 56), and NALP3 (57) has been reported. Interactions between mitochondrial membrane permeabilization and mtDNA release have also been described, with implications for disease pathogenesis, including myocardial infarction (58–61). Collectively, several studies have demonstrated the role of mitochondrial genes in regulating innate immune responses against bacterial infections (62, 63).

Mitochondria exhibit highly dynamic behavior collectively referred to as mitochondrial dynamics, which includes mitochondrial fission, fusion, mitophagy, and intracellular transport (64, 65). Dysregulation of these processes has been implicated in a wide range of pathological conditions and is also relevant in the context of bacterial infections such as pasteurellosis. In addition to the release of mitochondrial DNA (mtDNA) into the cytoplasm, recent studies have reported the transfer of intact mitochondria between cells, their presence in extracellular compartments, and even detection in blood plasma, suggesting a broader role for extracellular mitochondria in intercellular communication, mitochondrial dysfunction, and immune cell and platelet senescence (66, 67). In the present study, we identified key functional domains, including multiple haem-binding sites in cytochrome b and cytochrome c, which may contribute to their roles in cellular respiration and immune regulation (68). Similarly, important functional domains within NLRP3, including the NACHT and pyrin (PYD/DAPIN) domains, were identified, which are known to be critical for inflammasome assembly and immune signaling (69, 70). The Dual Role of Mitochondria in Infection as Immunity and its targets to Pathogen Manipulation have been well documented (71, 72). Dysregulation of these processes has been implicated in a wide range of pathological conditions and is also relevant in the context of bacterial infections such as pasteurellosis. In addition to the release of mitochondrial DNA (mtDNA) into the cytoplasm, recent studies have reported the transfer of intact mitochondria between cells, their presence in extracellular compartments, and even detection in blood plasma, suggesting a broader role for extracellular mitochondria in intercellular communication, mitochondrial dysfunction, and immune cell and platelet senescence (73, 74). In the present study, we identified key functional domains, including multiple haem-binding sites in cytochrome b and cytochrome c, which may contribute to their roles in cellular respiration and immune regulation (75). Similarly, important functional domains within NLRP3, including the NACHT and pyrin (PYD/DAPIN) domains, were identified, which are known to be critical for inflammasome assembly and immune signaling (76, 77). The Dual Role of Mitochondria in Infection as Immunity and its targets to Pathogen Manipulation have been well documented (78).

## 6 Conclusion

Collectively, these findings support the existence of a complex interplay between mitochondrial- and nuclear-encoded proteins in the context of bacterial diseases such as pasteurellosis. This coordinated interaction suggests that both mitochondrial and nuclear genes may serve as potential biomarkers of host immune status and disease susceptibility.

## Data Availability

The datasets presented in this study can be found in online repositories. The names of the repository/repositories and accession number(s) can be found in the article/supplementary material.
